# Protocol of a randomized controlled trial of the Tobacco Tactics website for operating engineers

**DOI:** 10.1186/1471-2458-12-335

**Published:** 2012-05-17

**Authors:** Sonia A Duffy, David L Ronis, Caroline Richardson, Andrea H Waltje, Lee A Ewing, Devon Noonan, Oisaeng Hong, John D Meeker

**Affiliations:** 1 Departments of Psychiatry and Otolaryngology, Ann Arbor VA Center for Clinical Management Research, The University of Michigan, School of Nursing, P.O. Box 130170, Ann Arbor, MI, 48113-0170, USA; 2 Ann Arbor VA Center for Clinical Management Research, The University of Michigan, School of Nursing, 400 North Ingalls, Ann Arbor, MI, 48109-5482, USA; 3 Department of Family Medicine, Fuller Building, 1018 Fuller Street, Ann Arbor, MI, 48104-1213, USA; 4 Clinical Research Coordinator, University of Michigan School of Nursing, 400 North Ingalls, Ann Arbor, MI, 48109-5482, USA; 5 Ann Arbor VA Center for Clinical Management Research, Health Services Research and Development, 2215 Fuller Rd., Ann Arbor, MI, 48105, USA; 6 Health Promotion/Risk Reduction Interventions with Vulnerable Populations, The University of Michigan, School of Nursing, 400 North Ingalls, Ann Arbor, MI, 48109-5482, USA; 7 Department of Community Health Systems, University of California: San Francisco (UCSF), 2 Koret Way, #N-531D, San Francisco, CA, 94143-0608, USA; 8 Environmental Health Science, School of Public Health, Environmental Hlth Science, M6017 SPH II, 1415 Washington Heights, Ann Arbor, Michigan, 48109-2029, USA

**Keywords:** Tobacco, Cessation, Smoking, Workplace intervention

## Abstract

**Background:**

Recent research indicates that 35 percent of blue-collar workers in the US currently smoke while only 20 percent of white-collar workers smoke. Over the last year, we have been working with heavy equipment operators, specifically the Local 324 Training Center of the International Union of Operating Engineers, to study the epidemiology of smoking, which is 29% compared to 21% among the general population. For the current study funded by the National Cancer Institute (1R21CA152247-01A1), we have developed the Tobacco Tactics website which will be compared to the state supported 1-800-QUIT-NOW telephone line. Outcome evaluation will compare those randomized to the Tobacco Tactics web-based intervention to those randomized to the 1-800-QUIT-NOW control condition on: a) 30-day and 6-month quit rates; b) cotinine levels; c) cigarettes smoked/day; d) number of quit attempts; and e) nicotine addiction. Process evaluation will compare the two groups on the: a) contacts with intervention; b) medications used; c) helpfulness of the nurse/coach; and d) willingness to recommend the intervention to others.

**Methods/Design:**

This will be a randomized controlled trial (N = 184). Both interventions will be offered during regularly scheduled safety training at Local 324 Training Center of the International Union of Operating Engineers and both will include optional provision of over-the-counter nicotine replacement therapy and the same number of telephone contacts. However, the Tobacco Tactics website has graphics tailored to Operating Engineers, tailored cessation feedback from the website, and follow up nurse counseling offered by multimedia options including phone and/or email, and/or e-community. Primary Analysis of Aim 1 will be conducted by using logistic regression to compare smoking habits (e.g., quit rates) of those in the intervention arm to those in the control arm. Primary analyses for Aim 2 will compare process measures (e.g., medications used) between the two groups by linear, logistic, and Poisson regression.

**Discussion:**

Dissemination of an efficacious work-site, web-based smoking cessation intervention has the potential to substantially impact cancer rates among this population. Based on the outcome of this smaller study, wider scale testing in conjunction with the International Environment Technology Testing Center which services Operating Engineers across North America (including US, Mexico, and Canada) will be conducted.

**Trial registration:**

NCT01124110

## Background

Blue-collar workers are at significant risk for cancer in that 35 percent are current smokers compared to 20 percent of white-collar workers [1]. Blue-collar workers are less likely to use proven tobacco cessation treatments compared to those of higher socioeconomic status (SES) [2]. In addition, blue-collar workers do not benefit from worksite smoking bans and restrictions. While there is an understanding of factors that contribute to elevated tobacco use in blue-collar workers, little research has focused on cessation. The data available suggests that developing novel approaches of disseminating efficacious interventions may be effective in reducing tobacco-related disparities and cancer among blue-collar workers [3].

Our preliminary data show that smoking rates are high among the Local 324 Training Center of the International Union of Operating Engineers at 29% compared to 21% among the general population [4]. The good news is that over half of the tobacco users reported that they are interested in quitting. With funds from the Department of Veterans Affairs (VA) and Blue Cross/Blue Shield of Michigan Foundation, we have built and pre-tested a Tobacco Tactics website and the results are promising. Hence, this funded National Cancer Institute R21 for Exploratory Grants for Behavioral Research in Cancer Control (1R21CA152247-01A1) is a randomized control trial (RCT) to test the Tobacco Tactics website intervention compared to the 1-800-QUIT-NOW quit line. The specific aims are to: Aim 1: Compare the efficacy of the Tobacco Tactics website intervention to the state sponsored 1-800-QUIT-NOW telephone line in improving cessation including: a) 30-day and 6-month quit rates; b) cotinine levels; c) cigarettes smoked/day; d) number of quit attempts; and e) nicotine addiction. Aim 2: Compare Operating Engineers randomized to the Tobacco Tactics website to those randomized to the 1-800-QUIT-NOW telephone quit line in terms of: a) contacts with the intervention; b) medications used; and c) helpfulness of the nurse/coach; and d) willingness to recommend the intervention to others (5 point scale ranging from strongly disagree to strongly agree).

### Risk of smoking among operating engineers

Among workers in dusty occupations, smoking is particularly detrimental to health because of the synergistic effect with occupational exposures which place workers at additional risk for respiratory disease [5]. Operating Engineers, those who operate heavy earth moving equipment, are particularly at risk for cancers of the lung [6], head and neck [7], and trachea and bronchus [8]. Since most Operating Engineers are men, unlike women, they may not seek regular health care. Yet even when seen by a health care provider, only 53% of construction workers were advised to quit smoking [9]. Given the high rates of smoking, the interaction between smoking and respirable dust exposure which enhances cancer rates, and lack of access to cessation interventions, an efficacious worksite smoking cessation intervention has the potential to substantially impact the health of Operating Engineers by reducing cancer rates.

### Cessation interventions

Countless RCTs have shown that cessation interventions that include both behavioral counseling and medications (nicotine replacement therapy and/or bupropion, or varenicline) are efficacious and produce quit rates ranging from 15–35% [10,11]. Studies have shown that the 1-800-QUIT-NOW telephone counseling programs offered in 48 states are efficacious although few smokers are reached [12]. A Cochrane Review of work site interventions concluded that group programs and nicotine replacement therapy increase quit rates, however, participation rates are low [13] .Only one worksite web-based intervention was found whereby 1,776 IBM employees were provided with the commercial QuitNet site showing a 43% quit rate among responders (13% intention-to-treat quit rate) [14], but this study did not include blue-collar workers, those most at risk for smoking.

Reports suggest that over 70% of all adults in the US are connected to the internet [15], 9.6 million blue-collar workers use the internet, and blue-collar workers are interested in computer technology [16]. While many smoking cessation websites are already available, many users encounter frustrations on sites which are poorly designed [17-19]. A Google search for “quit smoking” did not reveal some of the most efficacious cessation websites. Despite multiple design and informational problems, web-based cessation interventions have been shown to reduce tobacco use [20-23], be more efficacious than self-help booklets [24], be more efficacious if they provide tailored messages [25,26], and can enhance quit rates in conjunction with nicotine replacement therapy [24,25,27,28]. Thus far, no studies have compared web-based cessation interventions to the 1-800-QUIT-NOW telephone line.

Web-based interventions can also be enhanced with provider email or telephone contact [29]. A Cochrane Review meta-analysis showed that cessation advice by nurses increased the likelihood of quitting compared to advice without nurse counseling [30]. Studies have also shown that telephone counseling for cessation is efficacious [31,32]. Thus far, nurse-moderated web-based interventions have not been tested.

### Development of the Tobacco Tactics website for operating engineers

The efficacy of the face-to-face Tobacco Tactics intervention was tested among head and neck cancer patients in a published RCT and the face-to-face component has been disseminated to all inpatient smokers at two Veterans Affairs (VA) Hospitals compared to a control hospital [33]. Social marketing techniques were used to develop the image-based VA Tobacco Tactics program logo and campaign character [34]. Based on the face-to-face intervention, the Tobacco Tactics website was developed and pre-tested in the VA. Having found that smoking rates are high among Operating Engineers, we received a grant from the Blue Cross/Blue Shield of Michigan Foundation to redesign the Tobacco Tactics website for Operating Engineers.

### Theoretical framework

As in our prior studies, the theory guiding the Tobacco Tactics website intervention is the Health Belief Model [35]. This model proposes that behavior is influenced by Perceived Benefits (e.g., effectiveness of quitting smoking to reduce health risk and financial benefits), Perceived Barriers (withdrawal symptoms and other obstacles to quitting), Self-Efficacy (feeling of confidence that one can quit), Cues to Action (stimulus and reminders), by Perceived Susceptibility (chance of having negative health outcomes if continuing to smoke), and Severity of the Health Threat. Our current research has demonstrated that these characteristics are associated with 6-month quit rates and quit attempts.

## Methods

### Design

**Figure 1:**
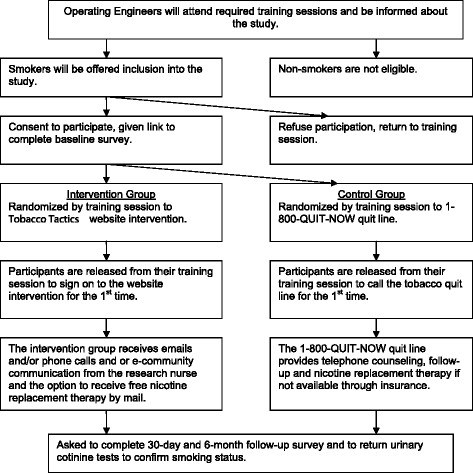
Experimental Design Overview.

The design for this two year study will be a RCT. The experimental group will receive the Tobacco Tactics website intervention. The control group will receive the 1-800-QUIT-NOW telephone quit line. Data on tobacco use will be collected at baseline, 30-day, and 6-month follow-up. See Figure 1 for a Consolidated Standards of Reporting Trials (CONSORT) diagram [36]. Institutional Review Board approval has been received from the University of Michigan.

### Setting/sample/power analysis

#### Setting

One of the greatest strengths of this proposal is the “buy in” that we have from leadership at the Local 324 Training Center of the International Union of Operating Engineers. While leadership has always been interested in the health of their workers, as evidenced by their prior participation in hearing protection studies, this “buy in” is even greater now that the trend in Michigan is to shift health care costs to unions [37,38]. Michigan Operating Engineers are required to attend annual safety training generally conducted at a centralized education center in Howell, MI. Some of the safety training is conducted face-to-face and some is conducted on the computer. Hence, we have worked with the Local 324 Training Center of the International Union of Operating Engineers to integrate the web-based Tobacco Tactics intervention into their annual safety training.

#### Sample

Inclusion criteria in this study are Operating Engineers who: 1) are attending a safety training course provided by Local 324 Education Center; 2) are greater than 18 years of age; 3) currently smoke; and 4) are interested in participating in a cessation program. A prior sample from this population has been described in another study [39]. In brief, the mean age was 43 (range18–70 years), most were male (92%), white (92%), married (68%), had a high school education or less (61%), and (29%) were current tobacco users.

#### Power analysis

For Aim 1, power analysis conducted with Power Analysis and Sample Size software (PASS) indicated that the sample size of 92 per group would provide 80% power to detect a 16% difference [33] in quit rates with alpha of .05 two tailed. For Aim 2 which will test of differences in means between the two groups, there will be 92% power to detect what Cohen [40] described as a medium sized difference when tested with alpha of .05, two tailed. Since Local 324 services about 16,000 Operating Engineers and about 29% smoke, we expect that we can easily recruit the 184 smokers needed for this study. To date 135 Operating Engineers have been randomized and recruitment and follow-up is ongoing.

### Procedures

#### Recruitment and pre-intervention survey

During a regularly scheduled safety training session, the study nurse will describe the study to participants who are told that it is voluntary. Those interested will be directed to a separate room and provided with an information pack which includes: 1) an introductory information letter; 2) consent form; 3) instructions for completing the baseline survey; and 4) instructions for accessing the intervention to which the session has been randomized. If they have any questions a member of the research team will be available.

If they choose to participate, they will sign one copy of the consent form and return it to the study team. Participants in both arms will be directed to an online baseline survey on Qualtrics, with questions about their tobacco use and other covariates described below. One of the questions will ask for the identification number on the participants’ handout to link the subject with their survey without storing personal identifiers.

#### Randomization and access to the intervention

Since there is a high probability for cross-contamination within training sessions, randomization will occur by training group (all individuals attending a specific training will be randomized to the same arm). The instruction sheet will give them information for logging on to the Tobacco Tactics website or calling the 1-800-QUIT-NOW telephone quit line. All subjects will be given time to make this first contact with the intervention during the training session where they will have access to a computer or telephone. After participating in the web-based Tobacco Tactics intervention, participants may continue to access the website from home, but this is not necessary as repeated access has not always been shown to increase efficacy of web-based interventions [25]. Both the intervention and control group will receive follow-up telephone calls. Both interventions are described in detail below.

#### Follow-up

Since return rates are lower for online surveys, to assess quit rates, we will use a two-step approach for collecting 30-day and 6-month follow up-surveys [41]. Subjects will initially be asked to complete the online survey. Those subjects who do not complete the online survey will then be mailed a paper survey to increase response rate. All subjects will also be mailed a NicAlert urinary cotinine test strip to confirm tobacco use status at the 6-month follow-up time point only (as the 30-day time point may give a false positive for smoking due to the use of nicotine replacement therapy). Participants in both study arms can receive a total of $50 remuneration (in the form of gift cards) for completion of surveys and cotinine tests.

### Description of Tobacco Tactics web-based intervention

#### General description

**Figure 2:**
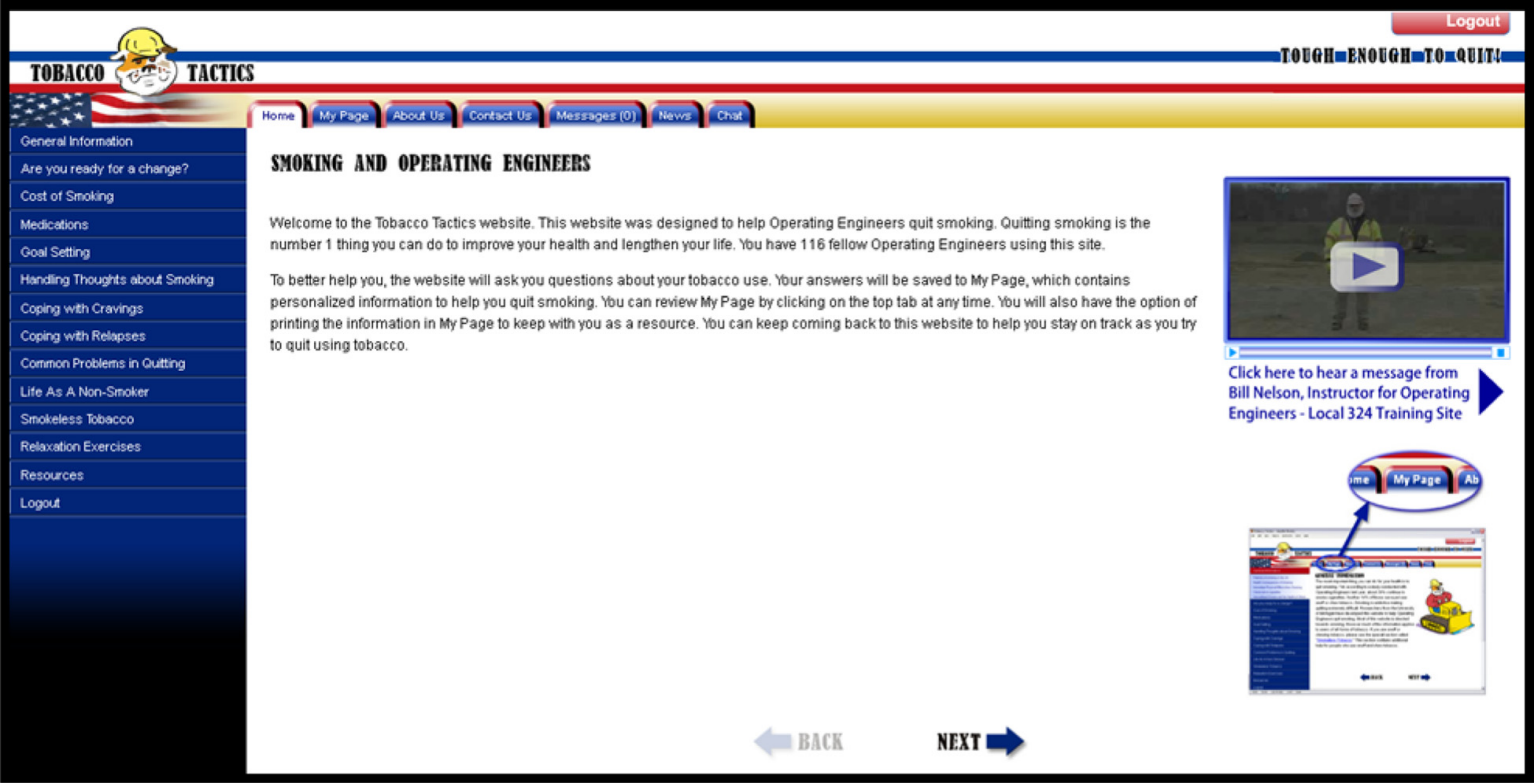
Sample Screen Shot of Tobacco Tactics Website.

The Tobacco Tactics website was developed based on a manual tested in a prior clinical trial and is in keeping with guideline recommendations for treatment of tobacco [42]. The content is written at an 8^th^ grade reading level. To ensure confidentiality participants are instructed to use their study identification (ID) as their user name. Participants create their own password at the time of first log-on. The home page is illustrated with colorful graphics and video of a testimonial from an Operating Engineer who quit smoking. The left side of the home page has “buttons” that link to modules that have been used in our Tobacco Tactics intervention. The top of the homepage has informational tabs. See Figure 2.

#### Preparing to quit

The left side of the homepage provides information on the perceived benefits and barriers to quitting. The General Information button provides content on the Patterns of Smoking among Operating Engineers, Health Consequences of Tobacco Use, Immediate Physical Effects from Cessation, Chemicals in Cigarettes including a scrolling list of the chemicals, and Second Hand Smoke. The Are You Ready for Change button provides interactive self-assessments on reasons for quitting, nicotine dependence, and identification of smoker type which then provide the Operating Engineers with tailored messages.

#### Medications

**Figure 3:**
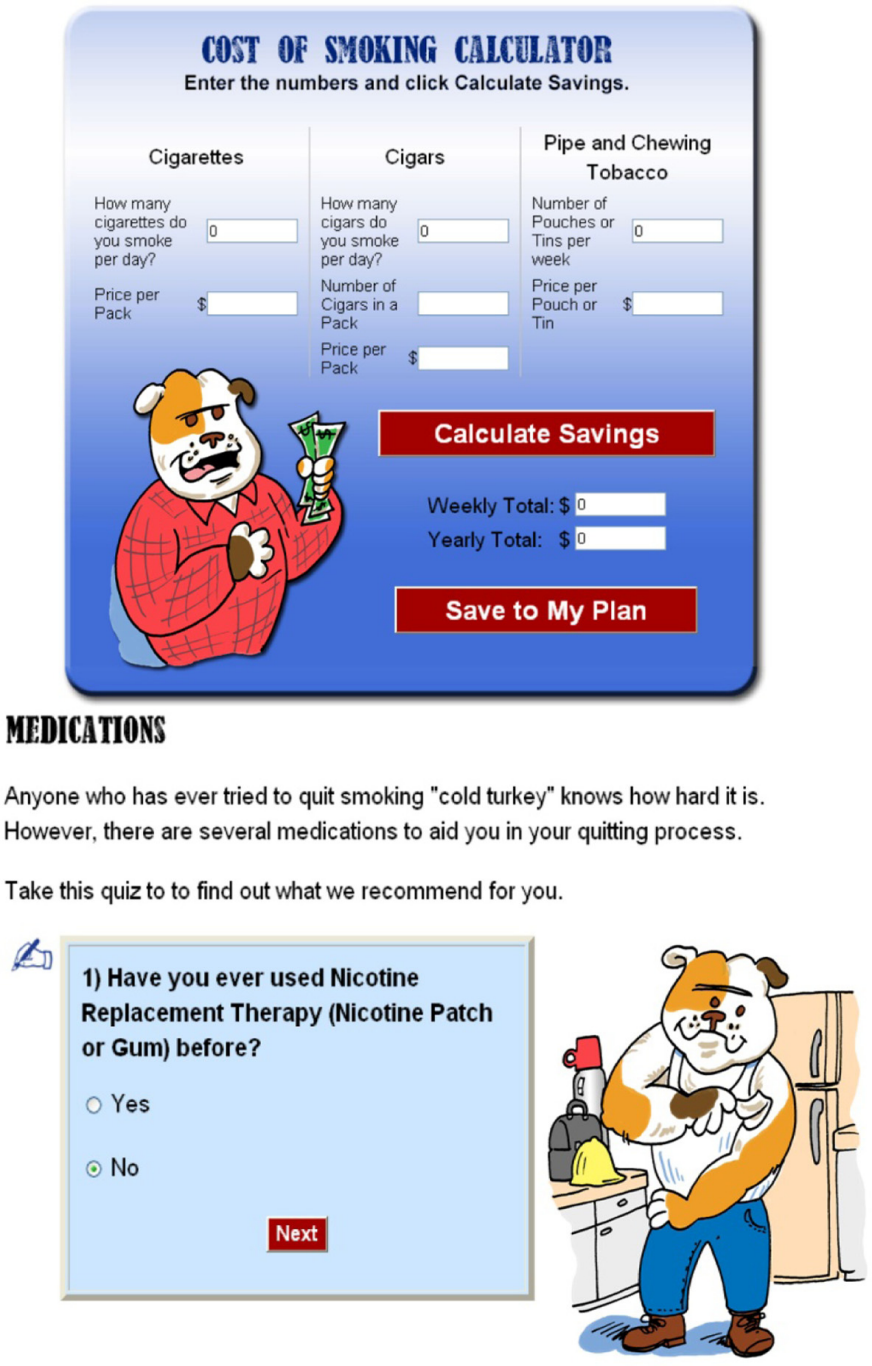
Sample Tobacco Tactics Web Pages.

Another button on the left side of the homepage links to a review of the medications available to assist with cessation and provides a suggested medication algorithm (enhancing self-efficacy). Operating Engineers will be offered their choice of a full supply of over-the-counter nicotine patch, gum, or lozenge. This is similar to what is done in other internet intervention studies [27], and is also done by the 1-800-QUIT-NOW quit line (control group). Those who have failed nicotine replacement therapy in the past will be encouraged to discuss prescription medications such as bupropion or varenicline with the study nurse who can assist with coordination of medications with their health care provider. See Figure 3 for additional screen shots.

#### Active quitting

Additional buttons on the left side of the homepage include information to enhance self-efficacy. The Goal Setting button has the Operating Engineer prepare for quitting by discarding all cigarettes, cleaning the car, informing family and friends, and setting a quit date. The Handling Thoughts About Tobacco button assists with assessing high risk situations and common triggers for relapse. The Coping With Cravings button gives tips based on their smoker type. The Coping With Relapses button discusses the role of alcohol, depression and relapse. The Common Problems in Quitting button discusses withdrawal symptoms and weight gain. The Life As A Non-Tobacco user button is designed to highlight how positive life can be as a nonsmoker. Additional buttons provide relaxation exercises and resources.

#### Informational tabs

“Tabs” across the top of the homepage include My Page which saves the participants’ data from interactive exercises. The tab About Us includes information about the study team. There is a tab to Contact Us. The Messages tab allows the study nurse to post messages for individual participants. The Latest News tab is a newsfeed from a smoking cessation blog. There is also a tab for an e-community.

#### Interactivity and tailoring

To enhance self-efficacy and perceived benefits and to reduce barriers, the treatments link will provide interactive cognitive behavioral therapy exercises tailored to the individual including a self-assessment of their tobacco habit, calculating a score about their level of addiction, identifying their smoker type, calculating money savings, preparing for quitting, (e.g., cleaning the car of cigarette butts, etc.), change plan work sheet, and coping with relapses. Additional interactive components will provide mechanisms for tobacco users to assess their smoking habit, set a quit date, and monitor weekly progress.

#### Nurse counseling

Since studies have shown that telephone and nurse counseling is efficacious [30-32] and tailored telephone and regular postal mail cessation interventions have been found to be efficacious among construction workers [43], the Tobacco Tactics web-based intervention will be enhanced with follow-up nurse telephone and/or email counseling contacts at 2, 7, 14, 21, and 30 days after the training. The follow-up contacts will reinforce the initial website visit, promote skill building, and monitor pharmacologic treatment. The nurse will not provide personal medical advice, but only expand on the information on the Tobacco Tactics website.

#### E-community/forum

Since peer support has been shown to enhance behavioral interventions [44], there will also be a nurse-monitored e-community to enhance self-efficacy. The nurse will serve as group moderator for the e-community and answer questions and post questions to stimulate group discussion. Each participant will be asked to select a pseudonym in order to establish an on-line identity while maintaining privacy. Following work by Lorig et al. [45], the e-community will be used to provide social support, both peer and professional. The e-community will allow intervention participants to post messages, ask questions and share success stories. While a disadvantage of the electronic forum is that the participant has to log in to both read and post messages, an advantage is that it will prevent e-mail overload and all postings can be monitored to prevent inappropriate messages or communication. The nurse will be trained to indentify messages deemed inappropriate, these messages will be deleted, the person that posted the message will be counseled and, if such behavior continues, they will have their access privileges revoked.

#### Informatics for the project

The Tobacco Tactics website for Operating Engineers was developed by Allen Wayne, LTD, who will also provide backup informatics during the course of the study. The website will be housed on a secure server at the University of Michigan.

### Control intervention

#### Principles of control group design

A recent article provides valuable insights into the design of control group conditions in clinical trials for behavioral interventions [46]. A fundamental principle is that the experimental and control conditions should be as equivalent on as many elements as possible including time spent, parallel recruitment periods and follow-up times, and attention given to participants. Both the intervention and control conditions should be of interest and value to the participants; control groups should not burden participants with worthless tasks as this may result in drop out of control group participants. Ethical considerations about withholding standard treatments should also be considered. When these conditions are met, the internal validity of the intervention is achieved.

#### Rationale for comparison arm

Given the principles of control group design, several options were considered as comparison arms for this study. One option was a “placebo” website (e.g., physical activity website); however, the literature shows that cessation websites work and we feel ethically obligated to provide some sort of cessation treatment. Another option was referral to another cessation website such as smokefree.gov, this being one of the better sites currently available; however, smokefree.gov is largely informational and has no interactive exercises or tailored messages and studies have shown that websites of this nature, while informational, are highly unlikely to engage participants and effect behavior change. A third option was to refer to an existing cessation treatment website; however, these sites often cost money and may not be well designed or marketed to appeal to Operating Engineers. After careful deliberation, we chose to compare the website to the 1-800-QUIT-NOW telephone quit line currently available in Michigan. The comparison arm was designed to answer the question that we felt would be of most value: Can the Tobacco Tactics website for Operating Engineers produce results better than an efficacious cessation treatment that is currently available in the community?

#### Description of 1-800-QUIT-NOW telephone quit line

The 1-800-QUIT-NOW quit line is a national program, run by each individual state, so the program can vary from state to state, however, we are only recruiting in Michigan so all participants will receive the same intervention. The first time someone calls the quit line, they will receive a personal coach who will assist them in setting a quit date and making an individualized quit plan. The personal coach also will provide on-going support with up to five telephone coaching sessions around the caller’s quit date. The first session usually lasts between 20–30 minutes and includes the intake evaluation and setting up appointments for the remaining counseling sessions. The length of the typical follow-up session is usually 20–40 minutes. Similar to our Tobacco Tactics intervention, the Michigan quit line provides free nicotine replacement therapy (patches or gum) for residents who are either uninsured or for those who have insurance that will not cover nicotine replacement therapy.

#### Equivalency across comparison groups

In keeping with the aforementioned principles of designing control groups, the Tobacco Tactics website and the 1-800-QUIT-NOW control conditions have been designed to be as equivalent as possible, in terms of time spent, parallel recruitment and follow-up, attention given to participants, and access to nicotine replacement therapy. Both groups are provided with a cessation intervention, nicotine replacement therapy, and equal numbers of follow-up contacts.

### Measures

#### Outcome evaluation (Aim 1)

To measure 30-day and 6-month cessation, we will ask participants in both groups if they have smoked cigarettes, even a puff, within the last 7 days [47]. Tobacco use status at 6 months will be confirmed by biochemical verification in the form of urinary cotinine test strips sent by mail. Harm reduction will also be assessed including cigarettes smoked/day, number of quit attempts, and nicotine addiction. Nicotine dependence will be assessed using the Fagerstrom Test for Nicotine Dependence (FTND) [48]. The Health Belief Model will be tested by questions (rated on a 5-point scale) used in our prior studies including: 1) Perceived Benefits: How important do you think quitting smoking is to your health? 2) Perceived Barriers: How difficult do you think it would be to quit smoking? and 3) Self-Efficacy: How confident are you that you will be able to stay off cigarettes? Based on current literature and pilot work done in this population, alcohol use [49], depression [50], stress [51], social support [52], comorbidities [53], and demographics [54,55] all may influence quit rates and therefore will be measured by valid and reliable instruments and controlled for as necessary in the analysis. Demographics have been shown to influence quit rates [54,55], so questions will be asked about age, gender, race, marital status, educational level, and veteran status.

#### Process evaluation (Aim 2)

For the tobacco users that are randomized to use the website, process evaluation will determine the: a) number of times they signed onto the website; b) medications used; c) number of nurse email/telephone calls per patient; d) helpfulness of the nurse/coach; and e) willingness to recommend the intervention to others (5 point scale ranging from strongly disagree to strongly agree) and satisfaction with the website. Similar survey questions will be asked of those randomized to the 1-800-QUIT-NOW telephone quit line intervention including: a) contacts with intervention; b) medications used; and c) willingness to recommend the intervention to others.

### Data analysis

The equivalence of the two groups will be tested using *χ*^2^ tests of association for categorical variables and t-tests for quantitative variables. Variables on which the groups differ will be included as covariates in the analyses. Clustering by group will be accounted for in analysis by using Generalized Linear Mixed Model variations of logistic regression, linear regression, and Poisson regression [56]. Analyses of all aims will be conducted by two-tailed tests with alpha of .05. An intent-to-treat approach will be used so that subjects are considered to be in the condition to which they were randomized despite how much they actually used that type of care.

The primary focus for Aim 1 is on comparing the two interventions (Tobacco Tactics website and 1-800-QUIT-NOW control condition) in their effects on smoking cessation using 6-month self-report together with results of the 6-month cotinine test. Using an intent to treat analysis, participants who cannot be reached or who fail to return the cotinine strip will be considered to be still smoking. Analysis of this measure will be conducted by using logistic regression of quit rate adjusted for clustering to compare the quit rates of those in the intervention arm to those in the control arm. Percentage quitting by group will be presented as a descriptive statistic to characterize the difference in quit rates between the two groups. This analysis will be supplemented by logistic regressions with smoking status as the dependent measure, treatment group as the independent variable, adjusted for clustering, but also adding in any baseline measures that were found in preliminary analyses to differ between groups, power permitting. If baseline measures differ between groups, we will effectively equate the groups. The same kinds of clustered logistic regression analyses will also be conducted on a less definitive version of the outcome measure: the self-report whether or not it is confirmed by a cotinine test. This analysis has the advantage of a slightly larger number of quitters identified because some people who actually quit smoking may not want to send in a urine sample (even on a cotinine test strip) because they fear it might reveal other problems.

Since tobacco use reduction is a feasible first step towards improved health, another approach is to look at smoking reduction less than quitting. These analyses will be conducted for those who continue to smoke and look at the number of cigarettes/day smoked, quit attempts, and nicotine addiction (FTND). The first two of these are quantitative measures available at baseline and at follow-up so will be analyzed by multiple linear regression with baseline measures included as covariates again adjusting for clustering in group assignments. Other covariates will be controlled as needed. Quit attempts is a count variable which will be analyzed by a Poisson regression (designed for analysis of counts) with treatment condition as the independent variable, controlling for covariates as needed. For all the measures analyzed to meet Aim 1 significant differences in quit rates and the quantitative measures between the Tobacco Tactics group and the 1-800-QUIT-NOW group will indicate whether the Tobacco Tactics intervention is more effective in influencing quitting.

Some of the process measures (e.g., number of times each module is accessed) for Aim 2 are distinct for the two interventions so will simply be analyzed descriptively. On the other hand most of the process measures (e.g., contacts, medications used, and ratings of helpfulness with the intervention) are comparable across groups so will be analyzed both descriptively, and compared statistically between the two groups. The distributions of these measures cover the range from counts (potentially Poisson distributed) to ratings (potentially normally distributed). Distributions will be examined before selecting the appropriate analytic method for comparison of groups.

## Discussion

The study design is novel in that, building on other studies that have compared websites to self-help materials or interactive versus non-interactive sites, this RCT rigorously compares the Tobacco Tactics website to the efficacious, “real world” 1-800-QUIT-NOW telephone quit line offered by the State of Michigan. The sample is novel in that to our knowledge, work-site web-based interventions have not been tested among blue-collar workers who have high tobacco use rates. The implementation strategy is novel in that it reduces barriers by incorporating the intervention into regularly scheduled computer-based safety trainings that Operating Engineers are already attending for their job, which will likely enhance participation rates. Coupling the website with nurse telephone and/or e-community counseling is novel in that the nurse can further enhance self-efficacy, clarify material, and trouble shoot difficulties with navigating the website, content, or medications.

The great advantage of the Tobacco Tactics website intervention is that it allows us to introduce Operating Engineers to cessation treatment during their regularly scheduled safety training. The intervention is particularly timely as the Michigan economy is depressed and unemployment rates are high, which results in increased stress, increased tobacco use, and increased risk for smoking related cancers among this population. Moreover, the new federal Affordable Care Act (ACA) contains numerous provisions to encourage prevention including worksite initiatives as most adults spend almost one-third of their time in the workplace.

Wide scale dissemination of an efficacious web-based smoking intervention has the potential to not only substantially impact cancer rates among this population, but also do so in a cost-effective manner. Leadership from Local 324 Training Center of the International Union of Operating Engineers is very excited about this project and has already discussed this project with leadership at the International Training Center which services North America (including the US, Mexico, and Canada). Based on the outcome of this smaller study, we will conduct wider scale testing and dissemination in conjunction with the International Training Center.

## Abbreviations

VA = Department of Veterans Affairs; RTC = Randomized Control Trial; CONSORT = Consolidated Standards of Reporting Trials; PASS = Power Analysis and Sample Size software; FTND = Fagerstrom Test for Nicotine Dependence; ACA = Affordable Care Act.

## Competing interests

The authors declare that they have no competing interests.

## Authors’ contributions

SD conceived of the study, and participated in its design and coordination and helped with drafting the manuscript. DR participated in the design of the study and performed the statistical analysis. AW and LE helped with drafting the manuscript and coordination of the study. DN helped with drafting the manuscript. All authors read and approved the final manuscript.

## Pre-publication history

The pre-publication history for this paper can be accessed here:

http://www.biomedcentral.com/1471-2458/12/335/prepub
